# Angiotensin-converting enzyme inhibitor-induced angioedema: Proposal for a diagnostic score^☆^

**DOI:** 10.1016/j.waojou.2025.101037

**Published:** 2025-03-12

**Authors:** Alexis Bocquet, Nicolas Marmion, Isabelle Boccon-Gibod, Laurence Bouillet

**Affiliations:** a Internal Medicine Department, National Reference Center for Angioedema/CREAK, Univ. Grenoble Alpes/CHU Grenoble Alpes, Grenoble, France; b Allergology Department/ CREAK, CHU Saint Pierre, Réunion, France

**Keywords:** Mast cell-mediated angioedema, Bradykinin-mediated angioedema, ACE inhibitors, ACE inhibitor-induced angioedema, Diagnostic score

## Abstract

**Objective:**

Angiotensin-converting enzyme inhibitor (ACEI)-induced angioedema (AE-ACEI) may be life-threatening, and the treatment should therefore be discontinued. However, patients taking ACEI may also have mast cell-mediated angioedema (AE-MC). Differentiating between AE-ACEI and AE-MC in patients taking ACEI is sometimes difficult. We propose to identify the factors associated with the diagnosis of AE-ACEI in patients.

**Materials and methods:**

A multicenter retrospective study was carried out at Grenoble Alpes University Hospital and University Hospital of La Réunion. All patients referred for suspected diagnosis of AE-ACEI were included in the study between January 2019 and January 2022. The final diagnosis was made by the expert physician after a minimum follow-up of 1 year and after a biological work-up ruling other bradykinin-mediated angioedema.

**Results:**

A total of 93 patients were analyzed, 49 with a final diagnosis of AE-ACEI and 44 with a diagnosis of AE-MC. Multivariate analysis identified 4 factors associated with the final diagnosis of AE-ACEI: number of AE between the introduction of ACEI and the consultation ≤3 (OR: 7.93 [1.60–50.7], p = 0.017) (1 point), duration of AE strictly greater than 24 h regardless of the treatments administered (OR: 8.41[2.07–44.5], p < 0.01) (1 point), hospitalization in intensive care unit (OR: 7.14[1.19–50.0], p = 0.045) (1 point) and no recurrence of AE after stopping ACEI, regardless of the delay (OR: 16.7[3.37–125], p < 0.01) (2 points).

This five-point diagnostic score (AUC: 0.85 [0.75–0.95]) identifies patients with a low probability of AE-ACEI when the score is 0–2 (sensitivity: 0.93, specificity: 0.35) and a high probability when it is between 4 and 5 (sensitivity: 0.53, specificity: 0.97).

**Conclusion:**

After a consultation in an angioedema expert center, the diagnosis of AE-ACEI has been excluded in almost half the patients. We identified a five-point score that could help in the diagnosis of AE-ACEI and in the decision to contraindicate the use of ACE inhibitors for life.

## Introduction

Angiotensin-converting enzyme inhibitors (ACEI) are widely and frequently prescribed treatments. Their benefits have been demonstrated in a wide range of diseases. They are recommended in particular for the treatment of chronic heart failure and arterial hypertension, and have been shown to have a nephroprotective effect.^1^^,^^2^

Of patients treated with ACEI, 0.1–1% experience angioedema (AE).^3^^,^^4^ Some of these adverse events are thought to be associated with an increase in bradykinin concentration, linked to a defect in bradykinin catabolism.^4^^,^^5^ Certain variants of the bradykinin receptor gene and the XPNPEP2 gene are thought to be associated with an increased risk of ACEI-induced AE (AE-ACEI). Like all bradykinin-induced angioedema (AE-BK), it can be life-threatening if not recognized and treated in time. If AE-ACEI is diagnosed, the drug should be discontinued permanently, otherwise AE attacks could happen again, with potentially more severe attacks.^3^

However, patients treated with ACEI may also develop mast cell-mediated angioedema (AE-MC).

Although the clinical features between AE-ACEI and AE-MC may be different, the diagnosis can sometimes be complicated, particularly in the absence of urticaria wheals, especially as no reliable biomarker currently exists to differentiate the 2 diagnoses.^6^

An incorrect diagnosis can have several consequences. In the case of a misdiagnosis of AE-MC, the continuation of treatment could lead to a life-threatening condition; and in the case of an misdiagnosis of AE-ACEI, discontinuing an effective treatment for chronic cardiovascular and nephrological conditions is done unnecessarily.

The aim of our study was therefore to identify the anamnestic and clinical factors associated with the type of final diagnosis (AE-ACEI and AE-MC) and then to propose a diagnostic score for AE-ACEI, using a cohort of patients referred for suspected AE-ACEI to 2 French angioedema centers of reference.

## Materials and methods

### Type of study

Multicenter retrospective study carried out at the Grenoble Alpes University Hospital and the University Hospital of La Réunion (CREAKs network).

The protocol was approved by the Grenoble Alpes University Hospital's Clinical Research and Innovation Department, which ensures compliance with good clinical practice and local ethics protocols. Patients could withdraw their consent or express their opposition to taking part in the study at any time.

### Population studied

All patients with suspected AE-ACEI from January 2019 to January 2022 with at least 12 months of medical follow-up and for whom a final diagnosis of AE-ACEI or AE-MC could be made, were included. Angioedema had to be isolated and not associated with urticaria wheals.

Each patient included in the study underwent a complete clinical history taken together with laboratory investigations. Theses investigations included, as a minimum, a C1 inhibitor assay (function and protein concentration) and a complement C4 assay, in order to rule out hereditary or acquired angioedema due to C1 inhibitor deficiency (respectively HAE-C1-INH and AAE-C1-INH), for which the use of ACEI would reveal the hereditary disease. In addition, a plasminogen or factor XII mutation test was performed in patients whose complement tests were normal and for whom there was a strong suspicion of HAE.

Patients with a follow-up of less than 12 months, with suspected angioedema caused by a drug other than ACEI, with non-isolated angioedema (presence of wheals), with an underlying diagnosis of HAE, with an uncertain diagnosis between AE-ACEI or AE-MC or who expressed opposition to participation in this study were excluded from the analysis.

### Data collected and primary endpoints

For each patient, the type of ACEI prescribed, the indication for the prescription, the patient's medical history of interest, in particular angioedema or atopy, demographic characteristics, clinical characteristics of AE attacks were collected. The treatments used and their efficacy, and any recurrence of angioedema attacks after stopping the ACEI were also collected.

For the primary endpoint, the definitive diagnosis of AE-ACEI or AE-MC was the diagnosis made 12 months after the initial consultation by the AE expert physician who had seen the patient.

### Statistical analysis and creation of a diagnostic score

Qualitative and quantitative data were described respectively by their numbers and percentages and by their median with their interquartile range (IQR).

In order to determine the association between the type of diagnosis and a clinical, anamnestic or therapeutic characteristic, a univariate analysis using logistic regression was carried out. Secondly, a multivariate analysis using logistic regression was performed with the variables of interest and the variables that were statistically significant in the univariate analysis.

A diagnostic score was compiled from all the characteristics statistically associated with the type of diagnosis in multivariate analysis and weighted according to their odds ratios.

Then, sensitivity, specificity with ROC curve analysis and area under the curve (AUC) were determined.

All analyses were done with R *(R Core Team (2021). R: A language and environment for statistical computing. R Foundation for Statistical Computing, Vienna, Austria).* A result was considered statistically significant when the p-value was less than 0.05.

## Results

**Fig. 1:**
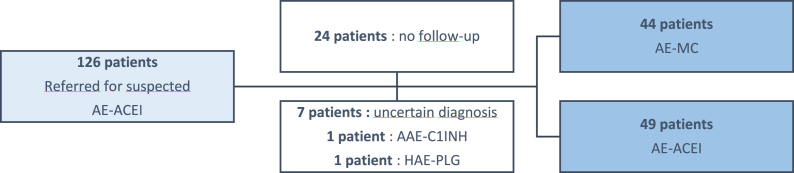
Flow chart. AE-ACEI: ACE inhibitor induced angioedema, AAE-C1-INH: acquired angioedema with C1-inhibitor deficiency, HAE-PLG: hereditary angioedema with plasminogen mutation, AE-MC: mast cell-mediated angioedema

From January 2019 to January 2022, 126 patients were referred for an episode of isolated AE suspected of AE-ACEI. Thirty-three patients were excluded including 1 patient with a diagnosis of AAE-C1-INH and 1 patient with a diagnosis of HAE with plasminogen mutation (HAE-PLG) (Fig. 1).

Ninety-three patients were included, including 44 patients (47.3%) with a final diagnosis of AE-MC and 49 patients (52.7%) with a final diagnosis of AE-ACEI. With a median age of 67 years (60–76), 58 were men (62.4%) and the median time between the introduction of ACEI and the first episode of AE on treatment was 36.5 months (3–60.8).

Table 1 summarizes the main characteristics of patients according to their final diagnosis.

**Table 1:** Final diagnosis and clinical features and outcomes for patients referred for a suspected AE-ACEI.

	AE-ACEI patients n = 49	AE-MC patients n = 44	Univariate Analysis *p*
**Sex ratio M/F,** n(%)	31/17 (65.3/34.7)	26/18 (59.1/40.9)	0.54
**Age (years),** median [IQR]	71 [65–78]	65 [58–71]	**0.018**
**Medical history,** n(%)			
Atopic disease	8 (16.3)	7 (15.9)	0.96
Acute urticaria	2 (4.1)	3 (7.0)	0.55
AE before ACEI introduction	3 (6.1)	9 (20.5)	0.05
**Time between ACEI introduction and first AE (days),** median [IQR]	1095 [75–3285]	1095 [112−1733]	0.08
**Localization of AE attacks**, n(%)			
Face	26 (54.2)	29 (65.9)	0.95
Tongue	29 (60.4)	26 (59.1)	0.90
Larynx	10 (20.8)	3 (6.8)	0.07
Abdomen	1 (2.1)	1 (2.3)	0.72
**Treatments,** n(%)	23 (46.9)	29 (65.9)	0.07
Antihistamine	0 (0)	27 (93.1)	
with Good efficacy	0 (0)	26 (96.3)	
Icatibant	23 (100)	9 (31.0)	
with Good efficacy	22 (95.7)	1 (3.4)	

AE: angioedema, ACEI: ACE inhibitor, AE-ACEI: ACE inhibitor induced angioedema, AE-MC: mast cell-mediated angioedema.

*p*-value is in bold when the result is statistically significant.

Patients with a diagnosis of AE-ACEI were older with a median age of 71 years (65–78) compared to patients with a diagnosis of AE-MC with a median age of 65 years (58–71) in univariate analysis (p = 0.018) but not in multivariate analysis.

Nine patients (20.5%) with AE-MC versus 3 patients (6.1%) with AE-ACEI had episodes of AE before the introduction of ACEI and 10 patients (20.8%) with AE-ACEI versus 3 patients (6.8%) with AE-MC had attacks of laryngeal localization. However, neither the existence of AE attacks before the introduction of ACEI nor the localization of the AE was associated with the type of AE. Similarly, gender, medical history of atopy, and the length of time between the introduction of ACEI and the first episode of AE under treatment were not associated with the type of final diagnosis.

Therapeutically, 23 patients with a diagnosis of AE-ACEI received icatibant. In 22 patients (95.7%), the treatment was reported to be efficient. In 27 patients with a diagnosis of AE-MC who received antihistamines during acute attack and sometimes the following day, 26 (96.3%) reported that the antihistamines were efficient.

### Factors associated with the final diagnosis of AE-ACEI or AE-MC

A number of AE less than or equal to 3 between the introduction of ACEI and the first consultation is associated with an AE-ACEI (in multivariate analysis, OR: 7.93 [1.60–50.7], p = 0.017). Similarly, a duration of angioedema strictly greater than 24 h (in multivariate analysis, OR: 8.41[2.07–44.5], p < 0.01) and hospitalization in an intensive care unit for the AE attack (in multivariate analysis, OR: 7.14[1.19–50.0], p = 0.045) are associated with a final diagnosis of AE-ACEI.

Finally, the absence of recurrence of angioedema after discontinuation of ACEI, regardless of the delay, was statistically associated with the diagnosis of AE-ACEI (in multivariate analysis, OR: 16.7[3.37–125], p < 0,01). Recurrence of AE after drug removal was observed in 23 patients (57.5%) with a diagnosis of AE-MC compared with 5 patients (10.2%) with a diagnosis of AE-ACEI. The median time to recurrence of AE for patients with a diagnosis of AE-ACEI was a maximum of 1 month (1-1).

All the factors associated with the type of final diagnosis are summarized in Table 2.

**Table 2:** Features and outcomes associated with the type of AE

			Univariate Analysis		Multivariate Analysis	
	AE-ACEI patients n = 49	AE-MC patients n = 44	OR	*p*	OR	*p*
**Age (years),** median [IQR]	71 [65–78]	65 [58–71]	0.96 [0.92–0.99]	**0.018**	NS	NS
**Number of AE since ACEI introduction,** n(%)						
>3	7 (14.3)	20 (45.5)	6.12 [2.18–18.9]	**<0.001**	7.93[1.60–50.7]	**0.017**
≤3	42 (85.7)	24 (54.5)				
**Duration of the AE,** n(%)						
≤24 h	15 (50.0)	31 (81.6)	4.43 [1.54–13.8]	**<0.01**	8.41[2.07–44.5]	**<0.01**
>24 h	15 (50.0)	7 (18.4)				
**Hospitalization in ICU**, n(%)	11 (22.9)	3 (6.8)	4.16 [1.16–20.0]	**0.042**	7.14[1.19–50.0]	**0.045**
**No AE attacks after ACEI discontinuation,** n(%)	5 (10.2)	23 (57.5)	11.9 [4.16–40.2]	**<0.001**	16.7[3.37–125]	**<0.01**

AE: angioedema, ACEI: ACE inhibitor, AE-ACEI: ACE inhibitor induced angioedema, AE-MC: mast cell-mediated angioedema, ICU: intensive care unit.

*p*-value is in bold when the result is statistically significant.

### AE-ACEI criteria diagnostic score

**Fig. 2:**
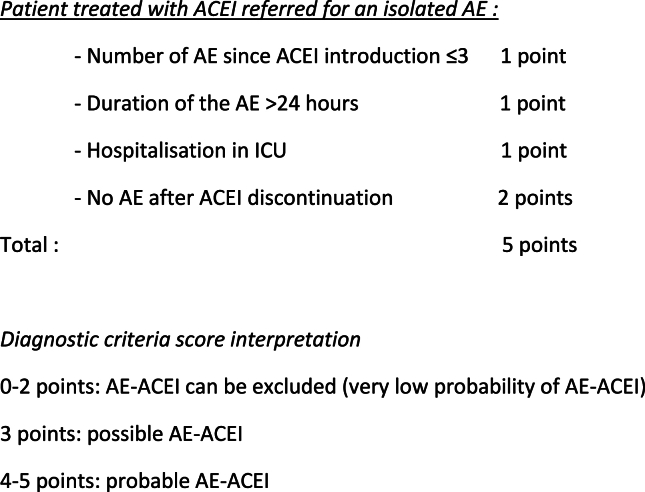
AE-ACEI Diagnostic criteria score. AE: angioedema, ACEI: ACE inhibitor, AE-ACEI: ACE inhibitor induced angioedema, ICU: intensive care unit

Weighted according to their odds ratios in multivariate analysis, the characteristics and outcomes associated with the type of angioedema are included in a proposed diagnostic score. This score is summarized in Fig. 2. It includes: a number of AE between the introduction of ACEI and the consultation ≤3 (1 point), a duration of AE strictly greater than 24 h regardless of the treatments administered (1 point), a hospitalization in intensive care unit (1 point) and no recurrence of AE after stopping ACEI, regardless of the delay (2 points). Greater weight is given to the absence of recurrence of AE after discontinuation of ACEI, given its odds ratio.

**Fig. 3:**
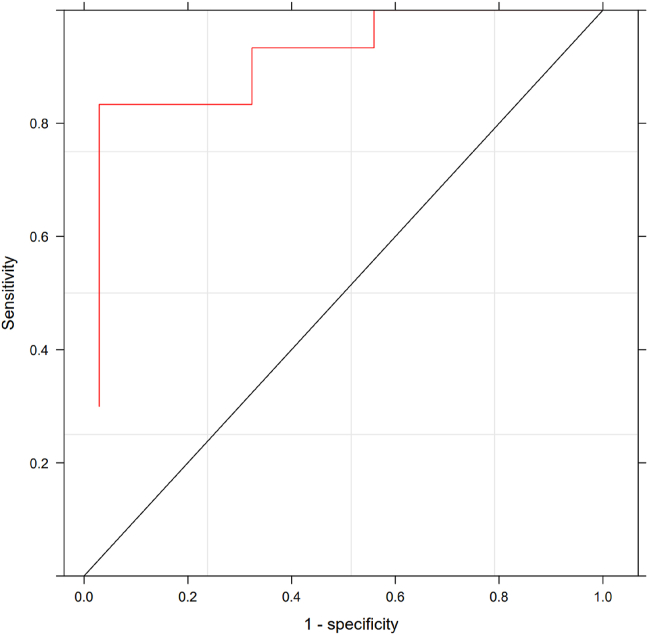
ROC curve of AE-ACEI diagnostic criteria score. AUC: 0.85 [0.75-0.95] AE-ACEI: ACE inhibitor induced angioedema

The ROC curve shows an AUC of 0.85 [0.75 0.95] (Fig. 3). With a score of 4 or more, there is a high probability of AE-ACEI, with a score specificity of 0.97 and a sensitivity of 0.53.

## Discussion

Our study showed that approximately 1 in 2 patients (47.3%) was refuted for the diagnosis of AE-ACEI after a follow-up by an AE specialist. These patients were therefore able to resume or continue their treatment with ACEI.

These results are comparable with the latest data in the literature, in particular those published in a French series by *Marie Douillard* et al in which the diagnosis of AE-ACEI was confirmed in 59% of patients.^7^^,^^8^

The factors associated with AE-ACEI are hospitalization in intensive care unit and the number of episodes less than or equal to 3 between the introduction of ACEI and consultation in a reference center. This can be explained by the severity of AE-BK compared with spontaneous AE-MC, which are classically benign.^9^, ^10^, ^11^, ^12^

The duration of angioedema strictly greater than 24 h is a factor associated with the final diagnosis of AE-ACEI. This is consistent with data in the literature, in particular the series by *Chepy* et al.^8^ Outside the context of ACEI treatment, it is accepted that AE-MC lasts for shorter periods than AE-BK, due to their different pathophysiology which is ultimately consistent with the association found in our study.^6^

Concerning the most discriminating criterion associated with the type of AE in our study, we were able to demonstrate that the absence of recurrence after discontinuation of ACEI favored the diagnosis of AE-ACEI. This finding did not include a specific time frame, whereas it had previously been accepted that recurrences of bradykinin-induced AE could occur up to 6 months after ACEI discontinuation. In our study, only recurrences within the first month after discontinuation of ACEI were observed. In an Italian series that followed 111 patients with a diagnosis of AE-ACEI over the long term, 46% of patients had a recurrence of AE attacks after stopping ACEI drug, 88% of which occurred during the first month of discontinuation. The absence of any change in the frequency of angioedema after discontinuation of ACEI, despite the change to another antihypertensive therapeutic class in this series, casts doubt on the actual bradykinin mechanism of AE in these patients, especially as the half-life of ACEI does not explain the persistence of bradykinin induced AE attacks 6 months after discontinuation.^13^

Finally, we could have chosen to include the criterion of recurrence of angioedema with a delay of 1 month in our score. In fact, patients with a diagnosis of AE-ACEI who had a new attack had it within 1 month of discontinuation.

Nevertheless, we have retained in our score the absence of a time limit for a new attack after discontinuation of ACEI, while defining more generally the presence or absence of a recurrence, in order to maintain a higher specificity of our score. In our opinion, specificity is the most important parameter in the hypothesis of new therapeutic studies.

In the end, our diagnostic score comprises 4 simple parameters, which can be assessed rapidly at the initial consultation and then at a single follow-up consultation. It gives clear importance to recurrence of AE following discontinuation of ACEI, and its AUC of 0.85 is interesting. Its specificity of 0.97 when the score is equal to 4 or 5 means that the diagnosis of AE-ACEI can be considered with certainty.

The advantage of this score is that it does not include a criterion concerning drug efficacy, although in our cohort, 95.7% of patients who received icatibant with a final diagnosis of AE-ACEI noted an efficacy of this treatment. Our diagnostic score, if confirmed in a larger cohort of patients, could therefore be used in a therapeutic trial to ensure the inclusion of patients with AE attacks whose mechanism is bradykinin and not mast cell-mediated. In the literature, and contrary to the results of our study which seem to confirm the efficacy of icatibant in patients with AE-IEC, the efficacy of this molecule blocking the B2 bradykinin receptor is subject to debate, with studies showing contradictory results.^14^^,^^15^ To explain this discrepancy, we may hypothesize that some patients included in the negative studies were in fact patients with a AE-MC and a misdiagnosis of AE-ACEI. In addition, the prescription of icatibant in real life in patients with angioedema on ACEI, particularly in France, seems to support the use of this compound, which has also led to French recommendations.^16^ The use of a diagnostic score to limit inclusion bias would seem to be of interest, and this is what we could propose with our score.

Unexpectedly, an improvement following icatibant administration in a patient with a diagnosis of AE-MC is reported in our study. This data should be treated with caution. Indeed, this patient had also received antihistamines. The imputability of efficacy of 1 molecule or another is therefore difficult, and has been attributed to both by default.

Other factors not included in the diagnostic score were the location of angioedema. In contrast to previous studies, the location of angioedema or the patient's medical history was not associated with a particular type of angioedema. Previous studies had shown that the location of the face outside the lips could be associated with AE-MC^7^ and also a history of atopy or allergy in another.^8^ This may be due to a lack of power, given the number of patients in our study. Nevertheless, the severity of angioedema due to a laryngeal location responsible for desaturation or asphyxia is indirectly present through the criterion of hospitalization in an intensive care unit. Overall clinical severity therefore seems to be a more important factor in the correct diagnosis than localization.

Interestingly, the presence of a history of AE prior to ACEI was not associated with the type of final diagnosis, which may be related to the size of our population. We can easily understand the presence of AE episodes for patients with a diagnosis of AE-MC, especially for those whose diagnosis will be chronic urticaria. As for the 3 patients with a history of AE before taking ACEI, we can hypothesize that they were induced by the release of histamine by mast cells, whether induced or spontaneous. The diagnosis of AE-ACEI does not exclude the possibility of mast cell-driven AE prior to the start of ACE inhibitor therapy. In fact, acute urticaria, some of which occurs only as AE, can affect up to 20% of the general population.^17^

With regard to the limitations of our study, this is a retrospective study of a cohort of patients which may include information bias. Nevertheless, there was little missing data, due to the fact that the patients were referred in expert centers’ familiar with AE, whether mast cell or bradykinin-mediated. Of course, this study is exploratory and the diagnostic score will need to be confirmed by a study testing its validity in a new and larger cohort of patients.

Moreover, the genetic search only included the 2 main genetic variants of HAE without C1-inhibitor deficiency, the factor XII and plasminogen variants. Some patients with other mutations may therefore have been included, but this is unlikely. Indeed, their prevalence is extremely low, and we can hypothesize that patients would have been classified as having an uncertain diagnosis in view of an associated family picture and persistence despite discontinuation of ACEI, and thus excluded from the analysis.^18^

In the end, our study shows that after a consultation in a AE center of reference, the diagnosis of AE-ACEI can be excluded in almost half the patients. This makes it possible to remove a contraindication to medication, a contraindication that could have significant consequences for patients if it were to be maintained. In addition, our study proposes a simple diagnostic score for AE-ACEI, requiring confirmation by another study and based on 4 simple criteria: recurrence after discontinuation of ACEI, the duration of AE, hospitalization in intensive care unit and finally the number of AEs since initiation of treatment and first consultation.

## Author consent for publications and contribution

All authors have contributed to conception and design of the study, data generation, analysis and interpretation of the data, preparation or critical revision of the manuscript.

All authors have approved the final version of the manuscript.

## Availability of data and materials

Aggregated and anonymized data are available.

## Funding

None.

## Declaration of competing interest

AB has consulted/served as speaker for, engaged in research and educational projects with or accepted travel grants from the following companies: BioCryst, CSL Behring, Takeda, Novartis, GSK.

NM: none.

IBG has consulted/served as speaker for, engaged in research and educational projects with or accepted travel grants from the following companies: BioCryst, CSL Behring, Takeda, Novartis, GSK, Blueprint, Kalvista, Pharvaris.

LB has consulted/served as speaker for, engaged in research and educational projects with or accepted travel grants from the following companies: BioCryst, CSL Behring, Takeda, Novartis, GSK, Blueprint, Kalvista, Pharvaris.

## References

[1] McDonagh T.A., Metra M., Adamo M. (Ed. June 2022). 2021 ESC Guidelines for the diagnosis and treatment of acute and chronic heart failure: developed by the Task Force for the diagnosis and treatment of acute and chronic heart failure of the European Society of Cardiology (ESC) with the special contribution of the Heart Failure Association (HFA) of the ESC. Rev Espanola Cardiol Engl.

[2] Alcocer L.A., Bryce A., De Padua Brasil D. (Nov 2023). The pivotal role of angiotensin-converting enzyme inhibitors and angiotensin II receptor blockers in hypertension management and cardiovascular and renal protection: a critical appraisal and comparison of international guidelines. Am J Cardiovasc Drugs Devices Interv.

[3] Montinaro V., Cicardi M. (2020). ACE inhibitor-mediated angioedema. Int Immunopharmacol. *Jan*.

[4] Agostoni A., Cicardi M., Cugno M., Zingale L.C., Gioffré D., Nussberger J. (15 Oct 1999). Angioedema due to angiotensin-converting enzyme inhibitors. Immunopharmacology.

[5] Nussberger J., Cugno M., Amstutz C., Cicardi M., Pellacani A., Agostoni A. (6 June 1998). Plasma bradykinin in angio-oedema. Lancet Lond Engl.

[6] Maurer M., Magerl M. (August 2021). Differences and similarities in the mechanisms and clinical expression of bradykinin-mediated vs. Mast cell-mediated angioedema. Clin Rev Allergy Immunol.

[7] Douillard M., Deheb Z., Bozon A. (August 2023). Over diagnosis of bradykinin angioedema in patients treated with angiotensin-converting enzyme inhibitors or angiotensin II receptor blockers. World Allergy Organ J.

[8] Chepy A., Veron M., Gautier S. (1 March 2022). Initial characteristics and follow-up of patients with a diagnosis of angiotensin-converting enzyme inhibitor induced angioedema. Allergy Asthma Proc.

[9] Bisinotto F.M.B., Seabra B.C., Lóes F.B.P., Martins L.B., Silveira LAM da (2019). [Postoperative angioedema induced by angiotensin-converting enzyme inhibitor: case report]. Braz J Anesthesiol Elsevier.

[10] Jackeviciute J., Pilvinis V., Pilviniene R. (august 2018). Fatal outcome of late-onset angiotensin-converting enzyme inhibitor induced angioedema: a case report. Medicine (Baltim).

[11] Atalay E., Özdemir M.T., Çiğsar G. (Dec 2015). Angiotensin converting enzyme inhibitor-related angioedema: a case of an unexpected death. Iran J Allergy Asthma Immunol.

[12] Stauber T., Confino-Cohen R., Goldberg A. (2014). Life-threatening angioedema induced by angiotensin-converting enzyme inhibitors: characteristics and risk factors. Am J Rhinol Allergy.

[13] Beltrami L., Zanichelli A., Zingale L., Vacchini R., Carugo S., Cicardi M. (Nov 2011). Long-term follow-up of 111 patients with angiotensin-converting enzyme inhibitor-related angioedema. J Hypertens.

[14] Baş M., Greve J., Stelter K. (29 Jan 2015). A randomized trial of icatibant in ACE-inhibitor-induced angioedema. N Engl J Med.

[15] Sinert R., Levy P., Bernstein J.A. (2017). Randomized trial of icatibant for angiotensin-converting enzyme inhibitor-induced upper airway angioedema. J Allergy Clin Immunol Pract.

[16] Rocour S., Cochard B., Daniel V., Martin L., Corvaisier M. (Jan 2023). Large predominance of off-label prescriptions of C1-inhibitor concentrates and icatibant in a real-life setting: a retrospective clinical study. J Clin Pharmacol.

[17] Riedl M.A. (2013 Sep-Oct). Hereditary angioedema with normal C1-INH (HAE type III). J Allergy Clin Immunol Pract.

[18] Kolkhir P., Giménez-Arnau A.M., Kulthanan K., Peter J., Metz M., Maurer M. (2022 Sep 15). Urticaria. Nat Rev Dis Primers.

